# Multi-state analysis of hypertension and mortality: application of semi-Markov model in a longitudinal cohort study

**DOI:** 10.1186/s12872-020-01599-7

**Published:** 2020-07-06

**Authors:** Azra Ramezankhani, Michael J. Blaha, Mohammad hassan Mirbolouk, Fereidoun Azizi, Farzad Hadaegh

**Affiliations:** 1 Prevention of Metabolic Disorders Research Center, Research Institute for Endocrine Sciences, Shahid Beheshti University of Medical Sciences, Tehran, Iran; 2 Ciccarone Center for the Prevention of Heart Disease, Johns Hopkins University School of Medicine, Baltimore, USA; 3 Endocrine Research Center, Research Institute for Endocrine Sciences, Shahid Beheshti University of Medical Sciences, Tehran, Iran

**Keywords:** Hypertension, Guideline, Multi-state, Mortality, Cardiovascular

## Abstract

**Background:**

Most previous research has studied the association of hypertension with cardiovascular disease (CVD) and all-cause mortality by focusing on the transition from the initial state to a single outcome. We investigated the impact of hypertension, defined according to the 2017 American College of Cardiology/American Heart Association (ACC/AHA) (new) and the Seventh Report of the Joint National Committee (JNC7) (old), on CVD death and all-cause mortality considering non-fatal CVD as an intermediate event between two CVD-free and mortality states.

**Methods:**

A total of 3002 Iranian population (47.4% men), aged ≥50 years were followed from 1999 to 2014. Two multi-state semi-Markov models with three transitions were defined for CVD death and all-cause mortality as two outcomes. The multivariable Cox model was used to estimate the effect of hypertension on transition hazards. The mean of 15-year life expectancy of participants in each transition was estimated using the restricted mean survival time.

**Results:**

The ACC/AHA guideline increased the prevalence of hypertension from 43.3 to 68.6%. Among CVD-free individuals, hypertension was significantly associated with increased risk of non-fatal CVD [Hazard Ratio, 1.52 (1.28–1.81) and 1.48 (1.21–1.80)], CVD death [2.96 (2.06–4.25) and 1.98 (1.30–3.04)] and all-cause mortality [1.64 (1.32–2.05) and 1.31 (1.01–1.69)] according the old and new guidelines, respectively. However, after incident non-fatal CVD, the association between hypertension and mortality events was not significant according to both definitions. Hypertensive participants experienced a first non-fatal CVD about 0.9 and 0.6 years earlier than normotensive population according to JNC7 and the 2017 ACC/AHA guidelines, respectively.

**Conclusion:**

Hypertension, according to JNC7 and the ACC/AHA guidelines, significantly increased the risk of mortality events among CVD-free population although the risk was attenuated using ACC/AHA guideline. Hypertension also decreased the number of years lived without CVD and early onset of CVD, and consequently, an increase in the time spent with these diseases. After non-fatal CVD, hypertension had no significant impact on mortality risk according to both guidelines.

## Background

By 2030, almost 23.6 million people will die of cardiovascular disease (CVD), mainly from coronary heart disease (CHD) and stroke [1]. There has been a well-established relation between hypertension and CVD as well as premature death [2, 3]. Considerable evidence exists about the impact of hypertension on the risk of CVD and mortality among CVD-free individuals [2, 4]. Moreover, a great number of studies has shown the effect of hypertension on recurrent CVD and all-cause mortality among individuals with history of CVD [5, 6]. The majority of these studies have focused on the transition from the initial state to a single outcome; however, patients may experience several events in the path between two initial and end points. For example, an increased risk of death among hypertensive patients after incident CVD has been evaluated without considering CVD as an intermediate event that take place “between initial condition and the endpoint”. Analysis in such studies is often performed using multi-state models (MSM) [7, 8]. In a MSM, a number of states are defined and the focus is on the process of going from one state to another [7, 9]. A widely used MSM, known as the illness-death model, can be used to evaluate whether previously diseased individuals have the same rate of death as those who have been healthy all their lives [8, 10, 11]. These models can provide a detailed insight into the effects of exposures on each state.

Recently, the American College of Cardiology/American Heart Association (ACC/AHA) [12] have proposed lower thresholds (> 130/80 mmHg) for blood pressure (BP) to define hypertension relative to prior guidelines (> 140/90 mmHg) [13]. Therefore, using the 2017 ACC/AHA and the Seventh Report of Joint National Committee (JNC7) criteria to define prevalent cases of hypertension, we undertook this study to investigate how MSM can be applied to study the effect of different definition for hypertension on the risk of CVD death and all-cause mortality with and without non-fatal CVD. We developed two MSMs each with three states: CVD-free (state 1), non-fatal CVD (state 2) and mortality events (state 3). In analysis of multi-state data, we focused on four topics: 1) the impact of hypertension on the probabilities of non-fatal CVD,CVD death and all-cause mortality among individuals free of CVD at baseline, 2) the probability of all-cause mortality and CVD death after non-fatal CVD occurrence, 3) life expectancy (LE) of participants in different states and comparing it among the participants with and without hypertension, and 4) the number of years of life lost due to hypertension with and without non-fatal CVD. To accomplish these goals, we analysed data from Tehran Lipid and Glucose Study (TLGS).

## Methods

### Participants

TLGS is an ongoing follow-up study of Iranian population with the goal of determining the risk factors and outcomes for non-communicable diseases [14]. Briefly, in phase 1 (1999–2001), about 15,000 individuals aged ≥3 years participated, and a total of 3550 new subjects were included in phase 2 (2002–2005). Upon entering the study, more examinations were conducted every 3 years. Besides the triennial reexaminations, all participants were followed up annually by telephone call about any medical event leading to hospitalization during the past year.

**Fig. 1:**
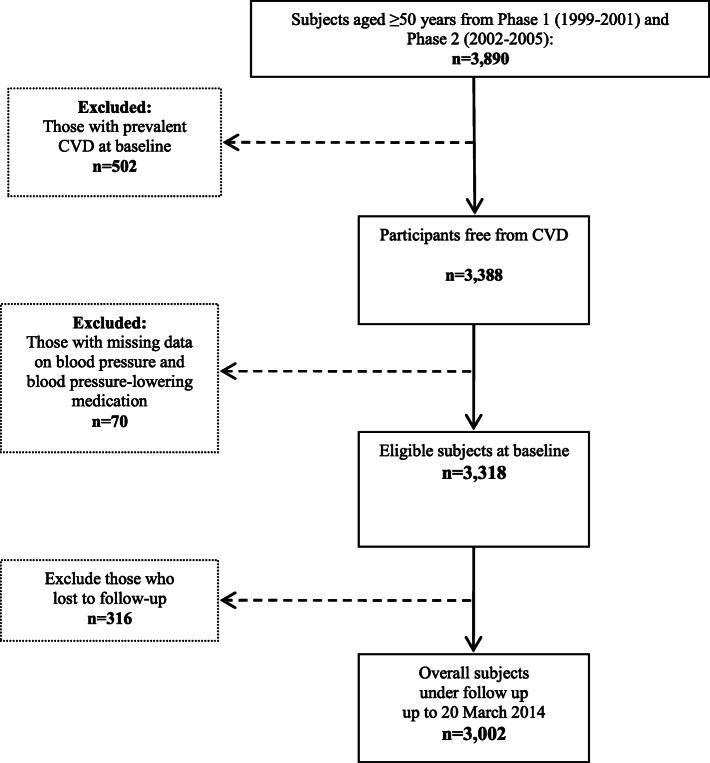
Study participants selection, Tehran lipid and glucose study (1999–2014)

In this study, all subjects aged ≥50 years (*n* = 3890) from the first and second phases were included. Participants with prevalent CVD at baseline (*n* = 502), with no information on hypertension status (*n* = 70) and without any follow-up data (*n* = 316) were excluded. The remaining 3002 (1422 men) participants were followed for different outcomes including non-fatal CVD and death from any cause (CVD and non-CVD death) until the end of the study (20 March 2014) (Fig. 1). The ethics committee of the Research Institute for Endocrine Sciences of Shahid Beheshti University of Medical Sciences approved the study and informed written consent was obtained from all participants.

### Data collection

In all examinations, participants completed a questionnaire on age, sex, history of CVD, medication use and smoking habits. Anthropometric measurements were also made, including height, and weight. After a 15-min rest in the sitting position, two measurements of BP were taken at the right brachial artery; the mean of two measurements was used to define systolic blood pressure (SBP) and diastolic blood pressure (DBP). Blood samples were obtained after a 12-h overnight fast to assess the fasting plasma glucose (FPG) and total cholesterol (TC) using standard laboratory techniques [14].

#### Exposure

In all examinations, hypertension was defined as a SBP ≥ 140 mmHg or a DBP ≥ 90 mmHg or taking antihypertensive medications according to the JNC7 guideline [13], and SBP ≥130 mmHg or a DBP ≥80 mmHg or taking antihypertensive medications in accordance with 2017 ACC/AHA guideline [12].

#### Confounding variables

We considered multiple potential confounders based on our previous study [15]. They included age, sex, TC, body mass index (BMI) calculated as weight (kg)/height (m^2^), smoking status (current, former/never), and diabetes mellitus (defined as FPG ≥ 7 mmol/L or 2-h post-challenge plasma glucose ≥11.1 mmol/L [16] or taking anti-diabetic medications).

#### Outcome

Details on the collection of outcomes in TLGS have been presented elsewhere [14, 17]. To summarize, in this study, all participants were followed annually for any medical conditions from entry into the study until the end of the study. The three outcomes for our analyses were incident non-fatal CVD, CVD mortality and all-cause mortality. Non-fatal CVD was defined as definite myocardial infarction (MI), probable MI, unstable angina pectoris, angiographic proven CHD, heart failure and stroke. CVD death was defined as fatal coronary artery diseases, MI, or stroke as either the primary or a contributing cause of death. All-cause mortality was defined as death from all causes including CVD and non-CVD death.

### Statistical analysis

Continuous and categorical variables were compared using the independent sample t-tests and χ^2^ test, respectively. Incidence density rate of events and respective 95% confidence interval (CI) were calculated by dividing the number of events by the person-years at risk. Missing data (after the exclusion criteria was applied) ranged from 0% to ~ 3% across the confounders; therefore, the multivariate imputation by chained equations (mice package in R software) [18] was implemented for handling missing data.

#### Multi-state model

**Fig. 2:**
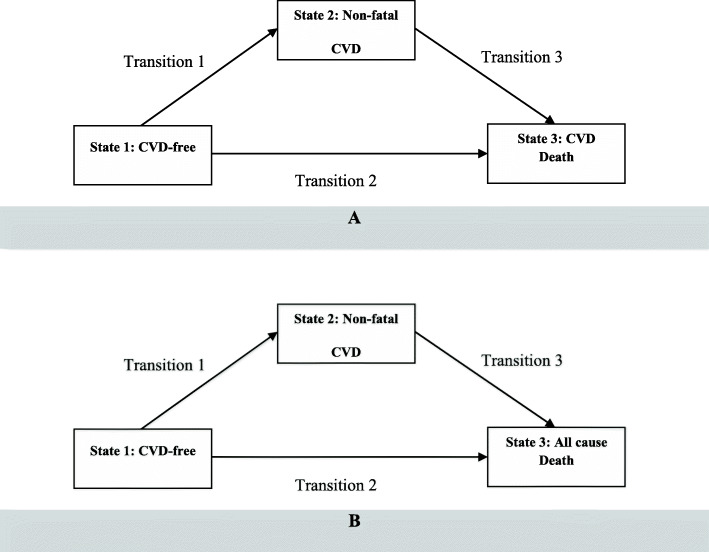
Graphical representation of the two multi-state models, **a** multi-state model 1 in which state 3 is CVD death, **b** multi-state model 2 in which state 3 is all cause death The association between hypertension, defined according to the 2017 ACC/AHA and JNC7, on CVD death and all-cause death considering non-fatal CVD as an intermediate event between two CVD-free and mortality states was estimated. ACC/AHA: The American College of Cardiology/American Heart Association. CVD: cardiovascular diseases. JNC7: The Seventh Report of the Joint National Committee.

We defined two MSMs for our data analysis (Fig. 2). In both MSMs, the state CVD-free was defined as state 1 and non-fatal CVD was defined as state 2. CVD death and all-cause death were defined as states 3 in MSM1 and MSM2, respectively. A change of state is called a transition. In Fig. 2, boxes represent the states and arrows show the possible transitions. All patients start in state 1, some of them move to state 2 (transition 1) and some patients transit directly to state 3 (transition 2). It is also possible to move from state 2 to state 3 (transition 3). State 3 (CVD death/all-cause mortality) is an absorbing state, because no transition can emerge from it [7]. This model is known as illness-death model [19]. The basic quantities of interest in a MSM are the transition intensities or hazard rates. The hazard rate is defined as instantaneous risk of moving from state ***r*** to state ***s*** at time ***t***. Two approaches often used in a MSM for definition of time ***t*** in the hazard function. The first approach is “clock forward” in which time ***t*** refers to the time since the individuals entered the initial state (start of study). In the second approach, defined as “clock reset”, time ***t*** refers to the time since entry of the present state. Thus, the clock is reset to 0 every time the patient enters a new state [9]. There are also different probability models to describe the way that an individual moves through a series of states in a multi-state process. A model that is often assumed in practice is the Markov model, which implies that the probability of going to a future state depends only on the present state and not on the history. The Markov property cannot hold when “clock reset” is considered as time scale; because, in this approach future state not only depends on the current state, but also on the entry time into current state. In case of “clock reset”, the resulting multi-state model is called a Markov renewal or semi-Markov model, which forms a sequence of embedded Markov models.

Finally, to estimate the hazard rates in a MSM, different functional forms such as parametric, semi-parametric or non-parametric approaches can be applied [9]. In our study, we used Cox’s proportional hazards model which also allows assessing the effects of multiple covariates on hazard rates. A preliminary analysis in both MSMs indicated independency of transition hazards going out from state 2 on the time at which that state was reached. Hence, a time homogenous semi-Markov model in the framework of Cox model was considered for data analysis.

To accommodate different baseline hazards for each transition, the data were stratified by transitions. If the endpoint of interest (states 2 and 3) had not yet occurred at the end of the observation period (20 March 2014), the event time was defined as right censored. The event time was calculated as the time between baseline examination and the event date (for event cases) or the last follow-up (for censored cases). Participants were censored due to death from a cause other than event of interest, loss to follow-up, or the end of the study without the event occurring.

All analysis was performed on a pooled sample wherein gender was included as a covariate in all models. The models were adjusted for age and sex, and were further adjusted for smoking status, TC, diabetes status and BMI. As we used “clock reset” to define the time scale in both MSMs, the time returns to zero at every transition, and the observations start at time zero after each transition. For illness-death models, this issue concerns only the transition from non-fatal CVD to mortality events. Therefore, for transition 1 and 2, the baseline measurements of exposure and covariates were used in MSMs. But, for the transition 3 (non-fatal CVD to mortality events), the most recent available values before incident of non-fatal CVD for each measurement was used. We also compared the magnitude of hazard ratios (HRs) of hypertension for two definitions of hypertension according to JNC7 vs. the 2017 ACC/AHA criteria in each MSM model.

#### Mean of survival time

We estimated the mean of 15-year LE of participants in each transition using the restricted mean survival time (RMST), defined as the area under the curve of the survival function up to a truncation time point τ (< ∞):

$$ {\mu}_t={\int}_0^{\tau }S(t) dt, $$

where S(t) is the survival function for the time T. The interpretation of μ in our study with a duration of 15 years is LE in each transition for the next 15 years [20]. The difference between hypertensive patients’ RMST and the RMST of the normotensive population was interpreted as the number of years of life lost due to hypertension [21]. We also estimated restricted mean time lost (RMTL) and the ratio of RMTLs for hypertensive and normotensive individuals in each transition. RMTL is defined as the average years of life lost during the follow-up time and is the area above the curve of the survival function up to a time t (*τ*-μ_t_). All analysis was done in R (https://CRAN.R-project.org). Unadjusted RMST and RMTL were estimated using survRM2 package and adjusted estimation for RMST were obtained by applying pseudo-value technique proposed by Kleinet al [22] using the pseudo package in R. The multi-state Markov model and the Cox PH model were fitted in R using the mstate [23] and survival packages, respectively. We used a *P*-value of < 0.05 (two tailed) to determine statistical significance.

## Results

### Descriptive

The cohort included 3002 participants between the ages of 50 and 88 years. The mean (SD) age of participants was 60.0 (7.5) years where 52% were women (Table 1). The prevalence of hypertension was 43.3 and 68.6% according to JNC7 and the 2017 ACC/AHA guidelines, respectively (Table 1). The reverse Kaplan-Meier estimate [24] of the median follow-up was 13.95 (interquartile range 10.29–14.50) years in MSM 1, and 14.09 (12.11–14.54) years in MSM 2. The numbers of transitions, both in terms of frequencies and percentages, are shown in Table 2. In both MSMs, 3002 individuals were at risk of transition from state 1 to states 2 and 3. Of these, 601(341 men) experienced a non-fatal CVD (transition 1) (Table 2). In MSM1, out of 601 individuals who experienced first non-fatal CVD, 71(51 men) died of CVD (transition 3) and 530 remained alive with non-fatal CVD until the end of the study. Among 3002 individuals free of CVD at baseline, 156 (96 men) died of CVD (transition 2). In MSM 2, from 601 individuals who experienced first non-fatal CVD, 126 (86 men) died of any cause (transition 3) and 475 remained alive with non-fatal CVD until the end of the study. Also, from 3002 individuals at the beginning of the study, 367 (210 men) died of any cause (transition 2) (Table 2).

**Table 1:** Characteristics of study cohort (*n* = 3002) at baseline according to JNC7 and the 2017 ACC/AHA guidelines for definition of hypertension, Tehran Lipid and Glucose Study (1999–2014)

	According to JNC7			According to the 2017 ACC/AHA			Total population ***N*** = 3002
	Hypertensive *n* = 1301	Normotensive *n* = 1701	P-value	Hypertensive *n* = 2060	Normotensive *n* = 942	P-value	
	***Mean (SD)***			***Mean (SD)***			***Mean (SD)***
**Sex**							
*Male*	531 (40.8)	891 (52.4)	< 0.001	897 (43.5)	525 (55.7)	< 0.001	1422 (47.4)
*Female*	770 (59.2)	810 (47.6)		1163 (56.5)	417 (44.3)		1580 (52.6)
**Age (year)**	61.4(7.6)	58.8(7.2)	< 0.001	60.5 (7.5)	58.9 (7.4)	< 0.001	60.0(7.5)
**TC (mmol/L)**	6.07 (1.28)	5.77 (1.18)	< 0.001	6.00 (1.25)	5.68 (1.17)	< 0.001	5.90 (1.23)
**SBP (mmHg)**	149.9(19.7)	119.1(11.4)	< 0.001	141.3 (20.0)	113.04 (9.5)	< 0.001	132.4(21.8)
**DBP (mmHg)**	88.8(11.5)	75.2(7.9)	< 0.001	86.01 (10.40)	70.46 (6.17)	< 0.001	81.1(11.7)
**BMI (kg/m**^**2**^**)**	28.8(4.5)	26.9(4.2)	< 0.001	28.4 (4.4)	26.2 (4.2)	< 0.001	27.7(4.5)
	***N (%)***			***N (%)***			***N (%)***
**Smoking status**							
*Current*	103(7.9)	293(17.2)	< 0.001	187 (9.1)	209 (22.3)	< 0.001	396(13.1)
*Never*	1033(79.4)	1211(71.1)		1620 (78.8)	624 (66.5)		2244(74.7)
*Past*	162(12.4)	192(11.2)		248 (12.1)	106 (11.3)		354(11.7)
**Diabetes**							
*Yes*	386(29.6)	294(17.2)	< 0.001	539 (27.0)	141 (15.5)	< 0.001	680(22.6)
*No*	872(67.0)	1353(79.5)		1456 (73.0)	769 (84.5)		2225(74.1)
**Antihypertensive medication use**							
*yes*	498 (38.3)	0		498 (24.2)	0		498(16.6)

JNC7, The Seventh Report of Joint National Committee

ACC/AHA, The 2017 American College of Cardiology/American Heart Association

*TC* total cholesterol, *SBP* systolic blood pressure, *DBP* diastolic blood pressure, *BMI* body mass index

**Table 2:** Numbers and percentages of population in the multi-state models; according to JNC7 and the 2017 ACC/AHA guidelines, Tehran Lipid and Glucose Study (1999–2014)

		Total entering	Destination states		
	Origin states		1: CVD-free	2: Non-fatal CVD	3: CVD death
**Multi-state model 1**	**1: CVD-free**	3002	2245 (74.8%)	601 (20%)	156 (5.2%)
	**2: Non-fatal CVD**	601	–	530 (88.1%)	71 (11.9%)
	**3: CVD death**	227	–	–	227 (100%)
**Multi-state** **model 2**			**1: CVD-free**	**2: Non-fatal CVD**	**3: All-cause death**
	**1: CVD-free**	3002	2034 (67.7%)	601 (20.1%)	367 (12.2%)
	**2: Non-fatal CVD**	601	–	475 (79.0%)	126 (21.0%)
	**3: All-cause death**	493	–	–	493 (100%)

In multi-state model 1, the state 3 is CVD death. Numbers and percentages of population in each transition are similar according to both JNC7 and the 2017 ACC/AHA guidelines

In multi-state model 2, the state 3 is all cause-death. Numbers and percentages of population in each transition are similar according to both JNC7 and the 2017 ACC/AHA guidelines

**Fig. 3:**
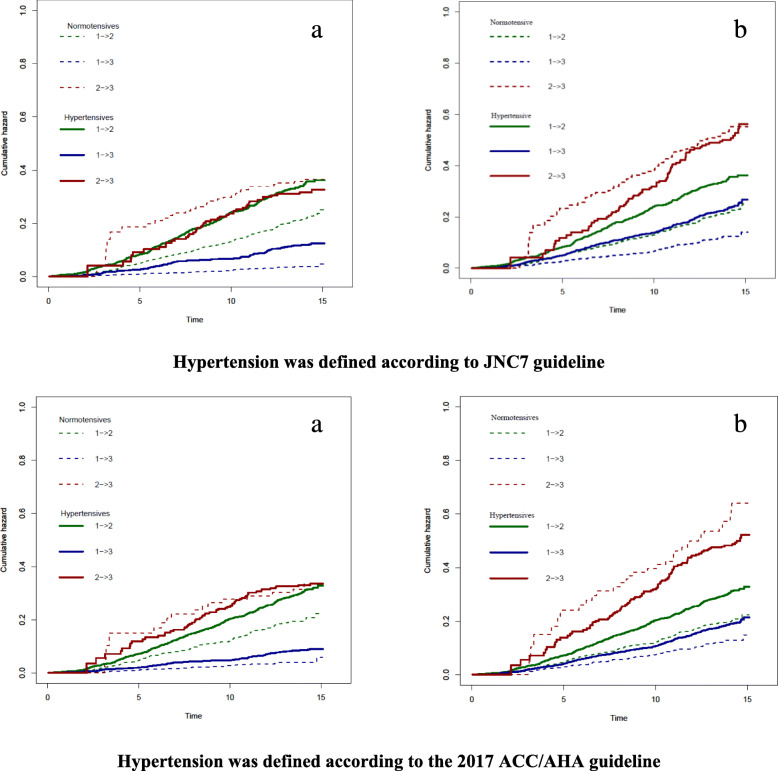
Estimated baseline cumulative hazards, normotensives vs. hypertensives, Tehran lipid and glucose study (1999–2014) **a** Multi-state model 1 in which the state 3 is CVD death. **b** Multi-state model 2 in which the state 3 is all-cause mortality. 1➔ 2: CVD-free to non-fatal CVD. 1➔ 3: CVD-free to CVD death in A, and CVD-free to all-cause death in B. 2➔ 3: Non-fatal CVD to CVD death in A, and CVD-free to all-cause death in B

The estimated cumulative hazards for the 3 transitions in two MSMs have been shown in Fig. 3. In both MSMs, the cumulative hazard is higher in transition 3 for normotensive participants.

### Results of MSM1

The results from MSM 1 are presented in Table 3. In confounders adjusted models, hypertensive individuals had 52 and 48% increased risk for non-fatal CVD according to JNC7 and the 2017 ACC/AHA guidelines, respectively, compared with their normotensive counterparts. The difference between the confounders adjusted HRs of hypertension according to JNC7 vs. the 2017 ACC/AHA guideline was not significant (*p* = 0.360).

**Table 3:** Estimation of Hazard ratios and confidence intervals of hypertension for non-fatal CVD and CVD death and all-cause death with and without CVD according to JNC7 and the 2017 ACC/AHA guidelines, Tehran Lipid and Glucose Study (1999–2014)

	Number of participants entered /number of events	Sex and age adjusted HR (95% CI)	Confounders adjusted HR (95% CI)	Number of participants entered /number of events	Sex and age adjusted HR (95% CI)	Confounders adjusted HR (95% CI)
**Multi-state model 1**						
**Transitions**	**According to JNC7**			**According to the 2017 ACC/AHA**		
**CVD-free to non-fatal** CVD						
Total	3002/601			3002/601		
Normotensive	1701/283	Reference	Reference	942/143	Reference	Reference
Hypertensive	1301/318	1.64 (1.39–1.93)^*^	1.52 (1.28–1.81)^*^	2060/458	1.55 (1.28–1.87)^*^	1.48 (1.21–1.80)^†^
**CVD-free to CVD death**						
Total	3002/156			3002/156		
Normotensive	1701/49	Reference	Reference	942/29	Reference	Reference
Hypertensive	1301/107	2.81 (1.99–3.97)^*^	2.96 (2.06–4.25)^*^	2060/127	1.92 (1.27–2.88)^*^	1.98 (1.30–3.04)^†^
**Non-fatal CVD to CVD death**						
Total	601/71			601/71		
Normotensive	245/30	Reference	Reference	142/20	Reference	Reference
Hypertensive	356/41	1.02 (0.63–1.64)	1.06 (0.65–1.72)	459/51	0.79 (0.47–1.33)	0.85 (0.50–1.44)
**Multi-state model 2**						
**CVD-free to non-fatal CVD**						
Total	3002/601			3002/601		
Normotensive	1701/283	Reference	Reference	942/143	Reference	Reference
Hypertensive	1301/318	1.64 (1.39–1.93)^*^	1.52 (1.28–1.81)^*^	2060/458	1.55 (1.28–1.87)^*^	1.48 (1.21–1.80)^*^
**CVD-free to all-cause death** CVD-free to CVD death						
Total	3002/493			3002/493		
Normotensive	1701/208	Reference	Reference	942/122	Reference	Reference
Hypertensive	1301/285	1.62 (1.31–2.00)^*^	1.64 (1.32–2.05)^*^	2060/371	1.30 (1.02–1.66)^†^	1.31 (1.01–1.69)^†^
**Non-fatal CVD to all-cause death**						
Total	601/126			601/126		
Normotensive	245/51	Reference	Reference	142/31	Reference	Reference
Hypertensive	356/75	1.09 (0.76–1.56)	1.13 (0.78–1.63)	459/95	0.95 (0.63–1.43)	1.00 (0.66–1.51)

In multi-state model 1 transition 3 is CVD death, and in multi-state model 2 transition 3 is all-cause death; Confounders included: age, sex, smoking, total cholesterol, prevalent diabetes and body mass index; CVD: cardiovascular disease; ^*^P-value< 0.001;^**†**^P-value< 0.01

Among 3002 participants free of CVD at baseline, hypertensive participants had 2.96 and 1.98 fold increased risk for CVD death according to JNC7 and the 2017ACC/AHA guidelines, respectively. Significant differences were found between the confounders adjusted HRs of hypertension according to JNC7 vs. the 2017 ACC/AHA guideline (*p* = 0.015). However, the risk of CVD death after the non-fatal CVD occurrence was not significantly associated with hypertension according to both JNC7 and the 2017 ACC/AHA guidelines (Table 3).

Table 4 shows the estimations of fifteen-year RMST and RMTL. Hypertensive participants were found to have 0.5 and 0.3 years shorter LE without CVD compared to normotensives according to JNC7 and the 2017 ACC/AHA guidelines, respectively. They also experienced a first non-fatal CVD about 0.9 and 0.6 years earlier than normotensive population according to JNC7 and the 2017 ACC/AHA guidelines. However, after experiencing a first non-fatal CVD, no significant difference in the LE of two hypertensive and normotensive groups was observed according to both JNC7 and the 2017 ACC/AHA guidelines. As shown in Table 4, the average years of life lost (RMTL) was 2.6 and 2.0 times higher in hypertensive vs. normotensive participants according to JNC7 and the 2017 ACC/AHA guidelines, respectively. Table 5 shows the results of confounders adjusted RMST. Hypertensive individuals had the shorter RMST, in transitions 1 and 2, according to both JNC7 and the 2017 ACC/AHA guidelines.

**Table 4:** RMST and RMTL stratified by transitions and hypertension status according to JNC7 and the 2017 ACC/AHA guidelines; Tehran Lipid and Glucose Study (1999–2014)

	Hypertension status	RMST Mean (CI)	RMST Difference (CI)	RMTL Mean (CI)	RMTL Ratios (CI)	RMST Mean (CI)	RMST Difference (CI)	RMTL Mean (CI)	RMTL Ratios (CI)
	Multi-state model 1								
**Transition**		**According to JNC7**				**According to the 2017 ACC/AHA**			
**CVD-free to non-fatal** CVD	Normotensives	13.6 (13.4–13.7)	−0.9 (−1.1,− 0.6)^*^	1.4 (1.2–1.5)	1.6 (1.4–1.9)^*^	13.7 (13.4–13.9)	-0.6 (− 0.8,-0.3)^*^	1.3 (1.1–1.5)	1.4 (1.2, 1.7)^*^
	Hypertensives	12.7 (12.5–13.0)		2.3 (1.9–2.4)		13.1 (12.9–13.2)		1.9 (1.7–2.1)	
**CVD-free to CVD death**	Normotensives	14.7 (14.6–14.8)	−0.5 (−0.7,− 0.3)^*^	0.3 (0.1–0.4)	2.6 (2.1–4.2)^*^	14.7 (14.6–14.8)	-0.3 (− 0.4,− 0.1)^*^	0.3 (0.1–0.3)	2.0 (1.3, 3.1) ^**†**^
	Hypertensives	14.2 (14.1–14.3)		0.8 (0.6–0.9)		14.4 (14.3–14.5)		0.6 (0.4–0.6)	
**Non-fatal CVD to CVD death**	Normotensives	10.6 (10.1–11.0)	−0.1 (− 0.7, 0.5)	1.4 (0.9–1.9)	1.1 (0.7–1.6)	10.6 (9.7–11.0)	-0.1 (− 0.5, 0.9)	1.4 (0.6–1.9)	1.1 (0.5, 1.4)
	Hypertensives	10.5(10.0–10.9)		1.5 (1.1–1.9)		10.5(10.2–10.9)		1.5 (1.1–1.8)	
**Multi-state model 2**									
**CVD-free to non-fatal CVD**	Normotensives	13.6 (13.5–13.8)	− 0.9 (−1.1,− 0.6)^*^	1.4 (1.2–1.5)	1.6 (1.4–1.9)^*^	13.7 (13.5, 13.9)	-0.6 (− 0.8,-0.3)^*^	1.3 (1.1, 1.5)	1.4 (1.2, 1.7)^*^
	Hypertensives	12.7 (12.5–13.0)		2.2 (1.9–2.4)		13.1 (12.9, 13.2)		1.9 (1.7, 2.1)	
**CVD-free to all-cause death**	Normotensives	14.2 (14.1–14.3)	−0.7 (−0.9,-0.5)^*^	0.8 (0.6–0.8)	1.9 (1.5–2.3)^*^	14.2 (14.0, 14.3)	−0.4 (− 0.5,-0.1)^**†**^	0.8 (0.6, 0.9)	1.5 (1.1, 1.8)^**†**^
	Hypertensives	13.5 (13.3–13.7)		1.5 (1.2–1.6)		13.8 (13.6, 13.9)		1.2 (1.0, 1.3)	
**Non-fatal CVD to all-cause death**	Normotensives	9.7 (9.2–10.3)	−0.3 (−1.1, 0.3)	2.3 (1.7–2.8)	1.1 (0.8–1.5)	9.51 (8.7, 10.2)	0.01 (−0.8, 0.9)	2.47 (1.8, 3.4)	1.01 (0.9, 1.1)
	Hypertensives	9.4 (8.8–9.8)		2.6 (2.1–3.1)		9.50 (9.1–10.0)		2.49 (2.0, 2.8)	

**RMST**: restricted mean survival time; **RMTL**: restricted mean time lost; In multi-state model 1 transition 3 is CVD death, and in multi-state model 2 transition 3 is all-cause death

**CI:** confidence interval; CVD: cardiovascular disease; **JNC7**: the Seventh Report of Joint National Committee; **ACC/AHA:** American College of Cardiology/American Heart Association. **RMST Difference:** shows the difference between RMST of hypertensives and normotensives; **RMTL Ratios:** is the ratio of RMTL of hypertensives to RMTL of normotensives

^*^P-value< 0.001;^**†**^P-value< 0.01

**Table 5:** Adjusted restricted mean survival time (RMST) of study participants according to JNC7 and the 2017 ACC/AHA guidelines; Tehran Lipid and Glucose Study (1999–2014)

	Difference of estimated RMST	SE	P-value	Difference of estimated RMST	SE	P-value
	Multi-state model 1					
**Transition**	**According to JNC7**			**According to the 2017 ACC/AHA**		
**CVD-free to non-fatal** CVD						
Hypertensives	−0.71	0.14	< 0.001	− 0.5	0.14	< 0.001
**CVD-free to CVD death**						
Hypertensives	−0.71	0.14	< 0.001	−0.5	0.14	< 0.001
**Non-fatal CVD to CVD death**						
Hypertensives	−0.12	0.4	0.75	0.10	0.40	0.838
**Multi-state model 2**						
**CVD-free to non-fatal CVD**						
Hypertensives	−0.71	0.14	< 0.001	− 0.54	0.14	< 0.001
**CVD-free to all-cause death**						
Hypertensives	−0.71	0.14	< 0.001	−0.54	0.14	< 0.001
**Non-fatal CVD to all-cause death**						
Hypertensives	−0.35	0.44	0.42	−0.30	0.44	0.567

In multi-state model 1 transition 3 is CVD death, and in multi-state model 2 transition 3 is all-cause death

**SE:** standard error; **CVD**: cardiovascular disease; **JNC7**: the Seventh Report of Joint National Committee;

**ACC/AHA**: American College of Cardiology/American Heart Association

Restricted mean survival times (RMST) were estimated using pseudo-value technique

Estimated RMST were adjusted for age, sex, smoking, total cholesterol, prevalent diabetes and body mass index

In multi-state model 1, patients with hypertension had the significantly shorter RMST, namely 0.71 in transitions 1 and 2, and 0.5 in transitions 1 and 2 according to JNC7 and the 2017 ACC/AHA guideline, respectively, than normotensive participants

In multi-state model 2, patients with hypertension had the significantly shorter RMST, namely 0.71 years in transitions 1, 2 according to JNC7 guideline and 0.54 years in transitions 1, 2 according to the 2017 ACC/AHA guideline, than normotensive participants

### Results of MSM2

Table 3 shows transition hazards from MSM 2. The results for transition 1 (CVD free to non-fatal CVD) was quite similar to the results of transition 1 in MSM1. In transition 2, the results of confounders adjusted models showed that hypertension was associated with 64 and 31% increased risk of all-cause death according to JNC7 and the ACC/AHA guidelines, respectively. The differences between two HRs was significant (*p* = 0.020). In transition 3, we did not find significant difference between hypertensives and normotensives regarding the risk of all-cause mortality after the non-fatal CVD occurrence according to both JNC7 and the 2017 ACC/AHA guidelines (Table 3).

Table 4 shows that the LE without CVD were 0.7 and 0.4 years shorter in hypertensives compared to their normotensive counterparts according to JNC7 and the 2017 ACC/AHA guidelines, respectively. Hypertensive participants experienced a first non-fatal CVD about 0.9 and 0.6 years earlier than normotensive population according to JNC7 and the 2017 ACC/AHA guidelines, respectively. No significant difference was observed between the LE of two hypertensive and normotensive participants after experiencing a first non-fatal CVD according to both guidelines. The RMTL were 1.9 and 1.5 times higher in hypertensive vs. normotensive participants according to JNC7 and the 2017 ACC/AHA guidelines, respectively (Table 4). The results of adjusted RMST showed that hypertensive individuals had the shorter RMST, in transitions 1 and 2, according to both JNC7 and the 2017 ACC/AHA guidelines (Table 5).

## Discussion

The results of multi-state analysis among CVD-free populations aged ≥50 years shows that although hypertension is significantly associated with transition to both CVD and all-cause mortality, using both the 2017 ACC/AHA and JNC7 guidelines, after incident non-fatal CVD (prevalent CVD), hypertension does not appear to have a significant association with either CVD or all-cause mortality according to both above mentioned guidelines. Moreover, hypertensive patients experienced a lower LE free of CVD and more years of life lost as compared to normotensive participants, with attenuation of this effect if a lower BP threshold was used via the 2017 ACC/AHA guidelines as compared to the JNC7. Also, this effect was primarily limited to the transition from CVD-free to either fatal events or non-fatal CVD events, and did not affect on transition from non-fatal CVD states to fatal events, using both guidelines.

Abundant epidemiological studies have shown the significant associations between hypertension and an increased risk of CVD/all-cause mortality [3, 25, 26]. More recently, we showed that hypertension increased the risks of CVD (HR: 1.89; 95% CI:1.20–2.98) and all-cause death (2.01, 1.26–3.20) among Iranian middle-aged population [3]. A meta-analysis showed that 20 mmHg higher SBP and 10 mmHg higher DBP were each associated with a doubling in the risk of death from CVD [27]. These findings have been confirmed in the present study, which reports 52 and 48% increased risks of non-fatal CVD according to JNC7 and the 2017 ACC/AHA guidelines, respectively. We also showed that among CVD-free population, hypertension was associated with increased risk of CVD death according to both JNC7 (2.96; 95% CI 2.06–4.25) and the 2017 ACC/AHA (1.98; 1.30–3.04) criteria; the corresponding values were (1.64; 1.32–2.05) and (1.31; 1.01–1.69) for all-cause mortality.

In fact, by lowering the cutoff values of SBP/DBP, the association between BP and mortality events among CVD-free population remained significant but was attenuated. It is quite evident that the more stringent BP thresholds will markedly increase the number of people classified as having hypertension [28–30]. As non-pharmacological therapy without taking medicines is recommended for all adults with SBP/DBP of 130–139/80–90 mmHg, excluding those with aged 65 years or older, pre-existing atherosclerotic CVD or a 10 year predicated risk of developing it of ≥10%, chronic kidney disease or T2D [12], the diagnosis of hypertension by using ACC/AHA guideline may provide an opportunity for people to change diet and lifestyle habits and to emphasize that BP is a risk factor that can be controlled [12, 31].

By using both JNC7 and AHA guidelines, we found no significant difference in the risk of CVD death and all-cause mortality between hypertensive and normotensive population after incident non-fatal CVD. However, using Framingham data in 2005, Franco and colleagues [32] arrived at different conclusions. In a multi-state life table analysis, they showed that hypertension was significantly associated with 29% increased risk (1.29; 1.10–1.52) of death after a non-fatal CVD. The loss of associations in transition 3 in our study might be attributable to the treatment of survivors of CVD by new therapeutic approaches. Also, Franco et al. [32] analysed data from 1948 to early 2000s, whereas we selected participants starting from 1999 and followed them until 2014, when there had been significant improvement in the management of hypertension after the incidence of CVD compared to the period between 1950s through 1980s. A number of interventional studies has consistently reported that the modification of potential risk factors [33] and the administration of beta-adrenergic-blocking agents [34] and aspirin [35] reduce the risk of adverse cardiovascular events after incident CVD. However, further investigations are needed to clarify and discuss our results.

We found that over 15 years follow-up, in MSM1,potential lifetimes were about 6 and 4 months fewer among CVD-free hypertensive individuals, according to JNC7 and the 2017 ACC/AHA guidelines, respectively, and in comparison to their normotensive participants. The corresponding values were 8.4 and 5 months for MSM2. Although few studies have investigated the impact of hypertension on LE in Western [32, 36] and Asian [37] populations, the effect of hypertension on LE has not been reported in the Eastern Mediterranean region with the high burden of CVD [38]. In the study conducted by Franco et al. [32], it was reported that at 50 years of age, total LE was 5.1 and 4.9 years longer for normotensive males and females, respectively, than for their hypertensive counterparts. In the present study, we found no significant difference in LE with CVD between hypertensive and normotensive individuals in both MSMs. However, Franco et al. [32] showed that hypertensives lived 2 years more with CVD compared with normotensive participants. A reason for the difference between their and our study could regard the different methodology used to calculate LE. While we estimated LE over 15 years of follow up using RMST, Franco et al. estimated total LE using life table analysis. Previous studies have suggested that the prognosis of survivors of non-fatal CVD is related to complex interactions between a number of factors, such as age, coexisting conditions, the extent of coronary artery disease, adjuvant medication use [39] and implementing effective interventions [40].

According to national and cohort studies in Iran, hypertension (defined by JNC7) was highly prevalent in adults [41–43]. In the last national study conducted in Iran, 25.2% (6.6 million cases) of Iranian people aged between 25 and 64 years had high BP, 45.5% were prehypertensive, 66% of hypertensive patients were unaware of their disease,75% did not take medication to lower BP and 76% had their BP uncontrolled [41]. These proportions will dramatically increase under the new ACC/AHA guidelines. Thus, applying new criteria could have a significant impact on individuals as well as on health care system. Although the new definition tends to increase acute health care costs, these treatments may delay the onset of a non-fatal CVD and incidence of mortality events and consequently other long-term care required.

This is the first multi-state analysis using Markov model which estimates the association between hypertension and mortality considering non-fatal CVD as an intermediate event. However, the results should be interpreted in the context of several limitations. First, due to the small number of CVD events, we were unable to investigate the cause-specific associations separately for each CVD event. Second, details about the treatment approach after incident non-fatal CVD were not available. Lastly, the study was conducted among Iranian population and therefore results might not be generalizable to other country.

## Conclusion

Using the 2017 ACC/AHA guideline increased the prevalence of hypertension in our population. Analyzing multistate data under a time homogeneous semi-Markov model showed that hypertension was associated with increased risk of non-fatal CVD, CVD death and all-cause mortality among CVD-free populations aged ≥50 years, and reduction in the number of years lived without CVD and early onset of CVD, and consequently, an increase in the time spent with these diseases. No significant association was observed between hypertension and mortality events after incident CVD using both JNC7 and 2017 ACC/AHA guidelines.

## Data Availability

All relevant data are included within the paper. Additional data are available upon request to the head of the RIES Ethics Committee, Dr. Azita ZadehVakili (azitavakili@endocrine.ac.ir).

## Abbreviations

ACC/AHA: The American College of Cardiology/American Heart Association
BMI: Body mass index
CHD: Coronary heart disease
CI: Confidence interval
CVD: Cardiovascular disease
DBP: Diastolic blood pressure
FPG: Fasting plasma glucose
HR: Hazard ratio
LE: Life expectancy
MI: Myocardial infarction
MSM: Multi-state model
RMST: Restricted mean survival time
RMTL: Restricted mean time lost
SBP: Systolic blood pressure
TC: Total cholesterol
